# Effects of a Life Skills Enhancement Program on the Life Skills and Risk Behaviors of Social Media Addiction in Early Adolescence

**DOI:** 10.2174/17450179-v19-e230113-2022-26

**Published:** 2023-02-16

**Authors:** Jedbordin Kumkronglek, Pornpen Sirisatayawong, Supat Chupradit

**Affiliations:** 1 Department of Occupational Therapy, Faculty of Associated Medical Sciences, Chiang Mai University, Chiang Mai 50200, Thailand

**Keywords:** Life skills, Life skills program, Social media, Social media addiction, Adolescence, Risk behavior, Cognitive behavior therapy and group therapy

## Abstract

**Objective:**

This research aimed to develop and investigate the effects of a life skills enhancement program on the life skills and risk behaviors of social media addiction in early adolescence.

**Methods:**

This research used a quasi-experimental design for a controlled study with a pre-test and post-test that collected data through a general information questionnaire, Social Media Addiction Screening Scale: S-MASS, and a life skills test. There were 48 samples recruited by purposive sampling from 5 schools in Chiang Mai, Thailand. The life skills enhancement program was developed under the theory of cognitive and behavioral therapy in combination with group therapy or occupational therapy. The program had a total of 10 sessions, with 1 session per week for 60 minutes and 10 weeks in total.

**Results:**

For the results, a statistically significant difference in post-test SMASS scores between the control and experimental group was found (p<0.01). Moreover, a statistically significant difference in the experimental group between pre-test and post-test using S-MASS scores decreased significantly after participating in the program but not in the control group. This result is similar to the comparative data of life skills scores that revealed there was a statistically significant difference between the pre-test and post-test only in the experimental group. For the comparative data between the control and experimental group, however, there were no statistically significant differences in pre-test and post-test life skills scores between the two groups.

**Conclusion:**

From the results, it can be summarized that the life skills enhancement program had affected an increase in life skills and a decrease in risky social media usage among adolescents.

## 1 INTRODUCTION

At present, social media has an enormous influence on human life.It is one of the activities that make our world connected so that we can communicate and do many things. In Thailand, 75% of the total population, which includes roughly 52 million people, use social media in their daily life, and this number will tend to increase by up to 4.7% in 10 months [1]. Adolescence is considered the group with the most social media usage in the population [2] because it can help them to meet their needs, such as having fun, exploring and expressing their identity, communicating with others online [3], and studying things that they are interested in so that they can have the ability to engage in meaningful occupations in the future [4]. Adolescents, especially those in early adolescence, often lack the life skills needed to restrain and control themselves from using social media. They tend to use social media excessively and this may affect their daily life activities, such as studying, sleeping, and socializing with family or friends in real life [5-7]. For these reasons, adolescents may be subjected to social media addiction [8]. Therefore, adolescents need to have the life skills that help them deal with their social media usage by recognizing their thoughts and emotions after managing them to make behavioral changes so that they can prevent social media addiction [9, 10]. At present, many studies have conducted life skills programs for different types of addiction groups with different theories or techniques [11-13]. However, there are currently no life skills programs that especially target social media addiction problems in the adolescent group. Social media addiction is currently a contemporary issue that is receiving a lot of attention from the perspective of a child and adolescent mental health, both in Thailand and abroad [1, 14]. As an occupational therapist, our researcher recognized the importance of occupational therapy’s role in the prevention and promotion of health, so we developed life skills enhancement programs based on the life skills and risk behaviors of social media addiction in early adolescence under the theory of cognitive and behavioral therapy in combination with the group process of occupational therapy.

## 2 MATERIALS AND METHODS

### 2.1 Research Objectives

The objectives of this research were to develop a Life Skills Enhancement Program for the life skills and risk behaviors of social media addiction in early adolescence and investigate the effects of the Life Skills Enhancement Program on life skills and risk behaviors of social media addiction in early adolescence.

### 2.2 Research Methodology

This research used a quasi-experimental design for controlled study with a pre-test and post-test. 48 junior high school students were recruited by purposive sampling from five schools located in an urban area of Chiang Mai, Thailand. After recruitment, 48 samples were obtained and evenly divided into two groups by stratified random sampling comprising 24 in the experimental group and 24 in the control group, as shown in Fig. (**1**). The G*power 3.1 test power analysis was used for calculating the sample size by using raw data from previous studies on CBT, Life Skills, and Addiction Problems in adolescence. The researchers used the sample size of the research by Du, Jiang, and Vance in 2010 [11] that was similar to this research sample and determined the sample size by a statistical power of 0.8 with a confidence level of 95%.

### 2.3 Data used in the Research

Tools of data collection had 4 parts as follows:

(1) A general information questionnaire was used for collecting data, including gender, the total number of siblings, current residence, co-residents, parents’ marital status, personality type, and length of time spent using social media in one day not related to school activities.

(2) The Social Media Addiction Screening Scale (S-MASS) was developed by Chanvit Pornnoppadol *et al*. In 2013 [15]. It measures adolescent behaviors related to social media use in the previous 3 months and classifies social media addiction into three levels, including low risk for 0-15 points, moderate risk for 16-30 points, and high risk of social media addiction for more than 30 points. S-MASS consists of 16 Likert-scale questions with a rating score from 0 to 3, 0 meaning not at all, 1 meaning probably not, 2 meaning probably true, and 3 meaning were absolutely true. Before using S-MASS for the data collecting phase, the researcher conducted Social Media Addiction Screening Scale trials with 30 junior high school students who were not sampled and found that Cronbach’s alpha coefficient was 0.887.

(3) A life skills test was developed by Amnat Rumruen and Porntip Chaiso in 2016 [16] for measuring the life skills scores of adolescents in 3 aspects, including cognitive skills, emotional skills, and social skills. It has 40 selective response questions about the students’ daily life situations with 4 choices which are the behaviors the students show in a given situation. Rating scores are1 and 0 points, with 1 point for a correct answer and 0 points for a wrong answer. This test had construct validity. Correlation coefficients between each component and total score were 0.65 - 0.81, while the item discrimination indices were in the range of 0.28 - 0.75, and the reliability of the test was 0.88.

(4) The life skills enhancement program was developed under cognitive and behavioral therapy in combination with the group process of occupational therapy [17]. The life skills components in this program are based on the life skills concept in an occupational therapy perspective consisting of cognitive skills, emotional skills, interpersonal, and social skills [18]. These three life skills components are relevant to the personality traits of adolescents at risk of social media addiction. In other words, adolescents at risk of social media addiction lacked these three skills, making them more likely to use social media excessively [19]. The structure of the program is based on the PEOP model, the occupational therapy model, to focus on dealing with the performance of people by enhancing life skills and making adolescents aware of the environment so that they can use it to reduce their social media usage. The Life Skills Enhancement Program was validated for content validity using the index of item objective congruence (IOC) by five specialists, including a mental health occupational therapist, mental health occupational therapy lecturer, child and adolescent psychiatrist and a specialist in psychology. Specialists must be qualified with at least 10 years of experience in a relevant field of work, and know about cognitive and behavioral therapy and the group process in occupational therapy. The life skills enhancement program also verified the validity of applying cognitive behavior therapy principles by a certified cognitive-behavioral psychotherapist by recording the program with 4 samples similar to the samples in the second phase of the research. This program contained a total of 10 periods with 1 session per week for 1 hour [12, 13]. The experimental group was divided into 3 groups with 8 people per group [20], while the control group received basic advice on how to manage their social media usage and had a normal life. The duration of the study was from November 2021 to March 2022. Activities in each session of this life skills enhancement program are shown in Table **1**.

Data analysis was evaluated by SPSS version 21. Descriptive statistical analysis was used for analyzing the results, including general information, S-MASS scores, and life skills scores. An independent t-test was used to identify the difference in S-MASS scores between the experimental and control groups at baseline and post-test. However, for identifying the difference in life skills scores between the experimental and control groups at the baseline and post-test, the Wilcoxon-Mann-Whitney Test was used. Paired t-test was used to analyze the differences in S-MASS scores between the pre-test and post-test in each sample group, while the Wilcoxon Signed Rank Test was used for analyzing the differences in life skills scores between the pre-test and post-test in each sample group. All analyses used *95*% *confidence intervals*, and the significance level was set at a p-value < .01.

## 3 RESEARCH RESULTS

### 3.1 General Information

There were 45 samples from all of them that completed the life skills enhancement program for 10 weeks (24 people for the experimental group and 21 people for the control group). The others dropped out from the control group because of personal reasons, such as needing to attend a cram school or not having the time to join. From the general information of the sample in the experimental group, it was found that a total of 58.33% (14 people) were female. Most of the samples had one sibling (70.83% or 17 people) and lived at their home (91.67% or 22 people) with a father and mother (66.67% or 16 people). Their parents’ marital status was married (66.67% or 16 people). For the samples’ personality types, 13 samples had extroverted personalities (54.17%), while the rest had introverted personalities. While the control group, it was found that a total of 57.14% (12 people) were female. Most of them had one sibling (76.19% or 16 people) and lived at their home (90.48% or 19 people) with a father and mother (61.91% or 13 people). Their parents’ marital status was married (61.91% or 13 people). 12 of them had extroverted personalities (57.14%), while the rest had introverted personalities, as shown in Table **2**.

### 3.2 Effect of the Life Skills Enhancement Program on Adolescent Risk Behaviors related to Social Media use among Early Adolescents (SMASS Scores)

For the results, the researcher compared SMASS scores for both pre-test and post-test samples in the program between the control and experimental groups. The analysis of comparative data on the pre-test and post-test of SMASS scores showed that there was no statistically significant difference in the pre-test between the control and experimental groups. For the post-test between the two groups, however, a statistically significant difference in the post-test between the control and experimental group was found (p<0.01), as shown in Table **3**.

The comparative data between the pre-test and post-test S-MASS scores in the experimental and control groups are shown in Table **4**. There was a statistically significant difference in S-MASS scores for the experimental group between pre-test and post-test as the S-MASS scores decreased significantly after participating in the program.

### 3.3 Effect of the Life Skills Enhancement Program on Life Skills among Early Adolescents (Life Skills Scores)

The comparative data of life skills scores on both pre-test and post-test samples of the program between the control and experimental groups showed that there were no statistically significant differences in pre-test and post-test between the two groups, as shown in Table **5**.

In terms of the comparative data for the pre-test and post-test life skills scores in the experimental group, there was a statistically significant difference between the pre-test and post-test (p < .01), as shown in Table **6**. It can be summarized that the participants had higher life skills scores after participating in the program, while the control group showed no statistically significant difference in the pre-test and post-test.

## 4 DISCUSSION

Researchers can use inferential statistics to analyze comparative data of both S-MASS scores and life skills scores. In terms of S-MASS scores, the experimental group had lower S-MASS scores than the control group at post-test with a statistically significant difference between them at the .01 level with a confidence level of .95. From the results, it indicates the effectiveness of the life skills enhancement program on risk behaviors of social media addiction; it helps samples to deal with their social media usage behaviors as they have less risky social media use behaviors and less obsession with social media after participating. This study result is consistent with previous life skills program studies conducted on behavioral addiction problems. It was found that the experimental group had less addictive behaviors and had better skills to manage their urge to use substances after participating in a life skills program under the theory of cognitive and behavioral therapy [11-13]. In addition, the data from previous studies of the life skills program also supported these results in that the life skills program helped adolescents to do their activities, that was, social media usage activity better by enhancing the necessary skills for observing and managing themselves [9, 10]. When comparing S-MASS scores between pre-test and post-test in the experimental group and control group, a statistically significant difference was found in the experimental group at the .01 level with a confidence level of .95, but not in the control group as the experimental group had lower S-MASS scores in post-test (19.21**±**7.157) than baseline (28.29**±**7.334). It can be summarized that this newly-developed life skills enhancement program that was structured based on the PEOP model, focusing on dealing and enhancing the performance of people, under the theory of cognitive and behavioral therapy in combination with the group therapy of occupational therapy has the effectiveness to significantly decrease the risky behaviors of adolescents in terms of social media usage and could be used for social media addiction problems in adolescence.

In terms of life skills, the researcher compared both the pre-test and post-test life skills scores between the two groups. The results showed that there were no statistically significant differences in pre-test and post-test between the two groups. However, the experimental group’s life skill score (28.50±4.809) was higher than the control group (24.86±7.101). As a result, it is not consistent with the previous life skills program study that showed the experimental group had significantly higher life skills than the control group after participating in the program [13, 21, 22]. From this result, it may be because of the activities in 8 and 9 lessons that specific for teaching social skills. These activities may be lack of practice and application of skills in daily life because these activities didn’t have behavioral homework for the samples to bring knowledge that they learned to use in their daily life. Therefore, it may not be enough to fully promote skills in these areas, which may affect overall life skills scores, resulting in life skills score that showed no statistically significant differences between two groups. However, a statistically significant difference (p < .01) was found in the pre-test and post-test for the experimental group when comparing the pretest and post-test life skills scores in the experimental group. It can be interpreted from these results that this life skills enhancement program is effective in promoting the life skills of the samples because the life skills scores increased significantly in the experimental group after participating in the program. For the effective use of the life skills program on life skills, however, it did not have strong effects on improving life skills from the interpretation of comparative data between the control and experimental groups. Although there were no statistically significant differences between the two groups, the life skill scores in the experimental group were still higher (28.50±4.809) than in the control group (24.86±7.101). Therefore, it can be concluded that this life skills enhancement program is effective to some extent in promoting life skills and can be used for improving the life skills of adolescents.

### 4.1 Limitations and Recommendations

In this research, we use the G*power 3.1 test power analysis to calculate the sample size. 48 samples were obtained, and this is a limitation of the study as the results of this study may not be used for references to larger populations. For future research, the study of a larger sample size needs to be conducted so that the results can refer to such populations.

As for the tools for measuring life skills, this study used a life skills test that was developed by Amnat Rumruen and Porntip Chaiso. This life skills test measures three life skills including cognitive skills, emotional skills, and social skills in everyday situations. Thus, it may be good if the tools can measure life skills used for specific problems and situations. For future research, researchers should develop and use a life skills test that is specific to social media use and addiction problems.

As for the activities in promoting social skills, this life skills enhancement program did not provide many experiences from behavioral homework for the samples to use skills that they learnt to apply to their daily life activities. Thus, for future research should adjust the form of activities and add behavioral homework to allow the samples to practice in their daily life even more.

Concerning recommendations for future research, study of the life skills enhancement program for social media addiction with a randomized controlled trial design should be conducted. Moreover, further research should be designed to compare the effects of a life skills enhancement program with other programs or other therapy for measuring the efficiency of the program.

## CONCLUSION

From the results, it can be concluded that this newly developed life skills enhancement program can be used for teaching essential life skills to adolescents at risk of social media addiction due to its effectiveness in significantly decreasing risky social media usage behaviors and increasing their life skills.

## Figures and Tables

**Fig. (1):**
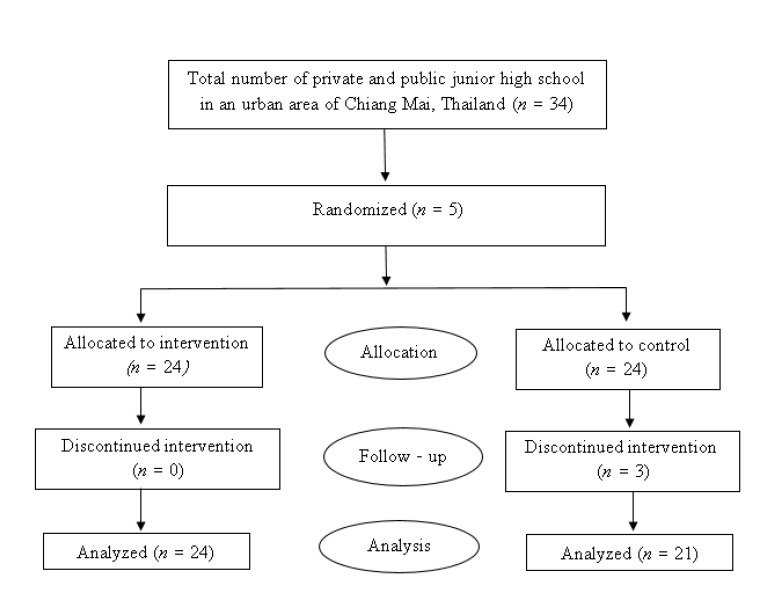
Consort flow diagram of the recruitment process.

**Table 1:** Activities in each session of life skills enhancement program.

**Activities**	**Techniques**	**Particulars**
1^st^ Lesson Let’s get to know social media addiction	Cognitive behavior therapy (psycho-education), group discussion, Questions and answers session.	Introduction and discussion about social media including definition and symptom, risk of social media addiction and benefits and harms of it. Lastly questions and answers session was conducted. This lesson aims to promote awareness of benefits and harms of social media and risk of social media addiction. Lastly, it aims to make adolescence realize the importance of controlling their social media usage behavior.
2^nd^ Lesson Dealing with your social media usage behavior	Cognitive behavior therapy (psycho-education), group discussion, presentation, feedback and behavior homework assignments.	How to use critical thinking skills effectively for solving social media usage problem in your daily life. This lesson aims to promote critical thinking and creative thinking skills, problem identification and problem solving skills.
3^rd^ Lesson Setting goals before taking action	Cognitive behavior therapy (psycho-education), group discussion, presentation, feedback and behavior homework assignments.	Learning about smart goal setting, self-monitoring, self-evaluation and self-reinforcement skills through activities and group discussion.
		This lesson aims to promote essential skills that contribute to the change in behavior and self-development including goal setting, self-monitoring and self-reinforcement skills.
4^th^ Lesson Knowing and understanding yourself	Cognitive behavior therapy (psycho-education), presentation and feedback.	Learning about your strength and weakness that may affect your self-control and goals. This lesson aims to promote self-awareness skills.
5^th^ Lesson Knowing your thoughts and emotions	Cognitive behavior therapy (psycho-education, affective education, thoughts awareness), group discussion, presentation, feedback and behavior homework assignments.	How to apply cognitive behaviors therapy theory to your daily life. Learning to observe your thoughts and emotions while you experience everyday events. This lesson aims to promote self-awareness skills including thoughts, emotions and behaviors.
6^th^ Lesson How to manage and deal with your emotions	Cognitive behavior therapy (psycho-education, affective education, diaphragmatic breathing), behavioral rehearsal, feedback and behavior homework assignments.	How to deal with your emotions or urge that make you always want to use social media. Learning about relaxing technique, including diaphragmatic breathing, progressive muscle relaxation and visual imagination. This lesson aims to promote your emotional management skills.
7^th^ Lesson How to deal with thoughts of using social media	Cognitive behavior therapy (psycho-education, cognitive restructuring), group discussion, presentation, feedback and behavior homework assignments.	How to deal with your thoughts that is the most important factor in behaviors therapy theory that cause urges to use social media. Learning about cognitive restructuring technique. This lesson aims to promote your thought management skills.
8^th^ Lesson Positive communication for good relationship	Cognitive behavior therapy (psycho-education), group discussion, presentation, Questions and answers session.	Positive communication for a good relationship. This lesson aims to promote effective communication, and interpersonal and social awareness skills.
9^th^ Lesson Making new friend	Cognitive behavior therapy (psycho-education), group discussion, presentation, Questions and answers session.	How to make a good relationship and maintain it. How to prepare yourself before communication with other people, moreover, how to monitor and deal with your thoughts and emotions while you speaks with other people. This lesson aims to promote critical thinking, relationship, interpersonal and social awareness skills.
10^th^ Lesson Important life skills that you learned	Cognitive behavior therapy (psycho-education), group discussion, presentation.	Cognitive skills, emotional skills and interpersonal and social skills, three essential life skills in the program, were summarized by subjects and researcher. After that, presentation and group discussion were conducted. This lesson aims to summarize and review essential life skills that were taught in the life skills enhancement program, moreover, apply them to adolescence’s social media problems.

**Table 2:** General Information of the samples (n = 45).

**-**	**Number **(%)	
	**Control Group** **(n=21)**	**Experimental Group (n=24)**
**Sex** Male Female	9 (42.86) 12 (57.14)	10 (41.67) 14 (58.33)
**Number of siblings** No siblings Have siblings	5 (23.81) 16 (76.19)	7 (29.17) 17 (70.83)
**Residence address** House Dormitory/Boarding house	19 (90.48) 2 (9.52)	22 (91.67) 2 (8.33)
**Currently living with** Both father and mother Father or mother Relative	13 (61.91) 7 (33.33) 1 (4.76)	16 (66.67) 6 (25) 2 (8.33)
**Parent's marital status** Together Divorce Separate	13 (61.91) 5 (28.89) 3 (14.29)	16 (66.67) 8 (33.33) 0 (0)
**personality type** Introvert Extrovert	9 (42.86) 12 (57.14)	11 (45.83) 13 (54.17)
**The amount of time spent on social media per day that is not related to school** 3 hours a day 4 hours a day 5 hours a day more than 5 hours a day	6 (28.57) 8 (38.1) 5 (23.81) 2 (9.52)	6 (25) 6 (25) 8 (33.33) 4 (16.67)
**Number of regularly used social media accounts** 2 Social Media Accounts 3 Social Media Accounts 4 Social Media Accounts 5 Social Media Accounts More than 5 social media accounts	8 (38.1) 9 (42.86) 2 (9.52) 2 (9.52) 0(0)	12 (50) 6 (25) 4 (16.66) 1 (4.17) 1 (4.17)

**Table 3:** Comparative data between the experimental group and control group on the pre-test and post-test S-MASS scores (n=45).

**Test**	**Experimental Group **(**n**=**24**)	**Control Group **(**n**=**21**)	**T**	**P**-**value**
	**Mean ± S.D.**	**Mean ± S.D.**		
**Pre-test**	28.29**±**7.334	28.38**±**5.652	-0.045	0.964
**Post-test**	19.21**±**7.157	29.81**±**6.660	-5.119	0.000**

**Note:** **p < 0.1

**Table 4:** Comparative data between the pre-test and post-test S-MASS scores in the experimental and control groups (n=45).

**Samples**	**Pre**-**test**	**Post**-**test**	**t**	**P**-**value**
	**Mean ± S.D.**	**Mean ± S.D.**		
**Experimental group (n=24)**	28.29**±**7.334	19.21**±**7.157	7.509	0.000**
**Control group (n=21)**	28.38**±**5.652	29.81**±**6.660	-1.303	0.208

**Note:** **pp < 0.01

**Table 5:** Comparative data on the pre-test and post-test life skills scores between the control and experimental groups (n=45).

**Test**	**Experimental Group **(**n**=**24**)	**Control Group **(**n**=**21**)	**U**	**P**-**value**
**Mean ± S.D.**	**Mean ± S.D.**
**Pre-test**	26.67±5.172	25.19±6.153	-0.434	0.664
**Post-test**	28.50±4.809	24.86±7.101	-1.748	0.080

**Table 6:** Comparative data on the pre-test and post-test life skills scores in the control and experimental groups (n=45).

**Samples**	**Pre**-**test**	**Post**-**test**	**Z**	**P**-**value**
	**Mean ± S.D.**	**Mean ± S.D.**		
**Experimental group (n=24)**	26.67±5.172	28.50±4.809	-3.134	0.002**
**Control group (n=21)**	25.19±6.153	24.86±7.101	-0.343	0.732

**Note:** **p < 0.1

## Data Availability

The data associated with the findings of this study are available on request from the corresponding author [S.C].

## LIST OF ABBREVIATIONS

S-MASS: = Social Media Addiction Screening Scale
IOC: = Item Objective Congruence
